# Enlistment of Seamen and Examination of Recruits

**Published:** 1853-07

**Authors:** G. R. B. Horner

**Affiliations:** Philadelphia, Surgeon U. S. Navy


*Enlistment of Seamen and Examination of Recruits.* By G. R. B. Horner, M. D., Philadelphia, Surgeon U. S. Navy.

The importance of this subject must be apparent to every reader, and I shall not enlarge upon it, but go on to speak of the most interesting parts. By the term seamen we mean all persons who enlist for the naval or merchant service, whether boys or men—mere landsmen, or sailors who have become more or less perfect in their trade. We also include in the term all persons serving in other capacities, on board ships of war or merchantmen, whether impelled by sails or by steam—and, we may now add, by heated air. By the term recruits we mean not only seamen of any grade, but marines, engineers, warrant and commissioned officers, &c., of any kind, who are subject to the inspection of surgeons and physicians, privately or publicly, for land or sea service, in our navy, army, volunteer and militia service. To every medical man the duty of inspecting such persons is liable to happen, and to him is left the decision, whether they be fit or unfit for service, either before or after enlistment; and he may be called on to reinspect recruits seeking discharge, or deemed mentally or corporeally unfit for service by their commanders. Frequently friends and relations may be the applicants for their discharge, and claim it on account of their ill health, when no other good reasons can be urged. It is very common for recruits to give most favorable accounts of themselves, to get enlisted, and, after admission into service, to become despondent, from loss of natural or artificial excitement—to regret what they have done, for various reasons, and feign or really deem themselves incompetent to discharge the duties required of them. The next act is to interest friends to get them discharged. To effect this many means are adopted, but none so willingly and conveniently as those intended to impose on the doctor, or to convince him that either he or some other medical man had overlooked certain defects at the recruit's admission into service. It becomes then necessary for the inspector to keep all his senses awake, to prevent imposition, professionally, upon himself, to do justice to those inspected, and above all to secure for public or private employment men who are competent in mind and body to discharge their duties. The passing of an incompetent person may please him and his friends, but injure the service in which he engages, and cause a fine to be imposed, much to the damage of reputation and mortification of the inspector, as in an instance related to me by a commander of our navy. An Englishman, shipped for the naval service, feigned lameness in one of the lower extremities, and to substantiate his assertions of disability—even before he got to sea—exhibited a huge scar upon it. A survey of him was ordered—the above and several other officers were ordered for that purpose—at Norfolk, and believing the man lame, they pronounced him "unfit for service," and recommended his discharge from it to the Secretary of the Navy. He accordingly directed it to be done, and the surgeon, most unjustly, to be mulcted in the sum advanced as pay, which is generally for three months. The order was promptly obeyed—the seaman was regularly discharged from the navy—a boat took him ashore, and no sooner had it touched the beach than he leaped on land, jumped into the air with joy and exultation, made a most insulting exclamation and gesture at the boat's crew, and ran into town as actively as any man in the ship could have done. One of my earliest recollections is of a like survey of a soldier in a militia regiment encamped at my native place, and on their way to defend the State against the invasion of the English, infesting with their fleet the lower counties of Virginia. This feigned invalid, however, was not so successful as the above. He had a very sagacious, old and experienced country physician, residing in the adjacent village, to deal with him. He was not to be fooled, and required ocular demonstration. The soldier, therefore, had to pull down his trowsers, to exhibit the rupture complained of. The doctor could find none—swore he was as sound as himself, and refused a certificate. However, the man got his discharge, though in a dishonorable way, for he was ordered to leave the camp, and was disgraced by being marched through the entire length of the village, with a drummer and fifer playing the rogue's or some other degrading march, and a crowd of boys hooting at his heels. To avoid similar impostures to the one first mentioned, and to be able to prevent the successful attempt of any individual making one like the second, or to keep out of employment persons physically unsuited for it, I will now mention the general rules adopted by myself and others, and which I recommend after the inspection of more than two thousand persons within the last eight years, without counting many straggling cases examined by me while not engaged regularly in the recruiting service, and likewise exclusive of many hundreds inspected anterior to that time, in active service, and while attached to the U. S. Rendezvous in 1834, '35 and '36.

To secure the government against the introduction of physically unfit persons into the naval service, the rule adopted there is for the officers of the line to refer them for physical examination to the surgeon, after they have been found in other respects suitable. If he pass them, he gives printed certificates of his having found them fit for the naval service, and on the same one writes a description of them, viz.: of their "birth-place, age, color of the eyes and hair, complexion, trade, height, marks of vaccination, &c., and the number of years they have been at sea and in service." When the surgeon has finished these certificates and signed them, one of the other officers fills up the blank orders at the foot, for the recruits being taken on board the receiving vessels. They are then, if sailors, generally given several days' liberty, and retire with their landlords, after these have taken the certificates and become their security. For this and money advanced they charge a large per centage. The recruits having had their frolic, if sober enough, are, at the expiration of their liberty, delivered on board, with the clothing and bedding required, and re-examined by one of the medical officers attached to the station. To avoid any ill feeling which might result from a recruit's being rejected by him, the junior medical officer, Passed Assis't Surgeon J. O'C. Barclay, first re-examines, and if he should find the recruit unfit for service, is required to return him to the Rendezvous and report the defect found. Should the surgeon there still deem him fit for service, the recruit has to be referred to myself, who give the casting vote after a third examination. But such is the strictness of the first one, that this is rarely necessary; and it is surprising, considering the excesses into which the recruits engage before their delivery on board.

For the marines enlisted on this station there is only one examination, which is made by myself or assistant, according to the time of the day when they present themselves. But all the candidates—as officers of the Engineer Corps—who attended the late examination here, had first to be inspected by me, and to get their orders endorsed with a certificate of their having been examined and passed physically. Of the first I made a general examination, with as little exposure of the person as possible, as they all had a gentlemanly appearance; but having understood from a member of the Board of Engineers that one had been at a former session rejected for rupture, I was more strict with the last examined, and in addition to a written declaration of their soundness, which was read to them for their assent, I omitted nothing thought requisite to detect important defects, though on examination of the above candidate no rupture could be found; and he only had an unnatural depression of the sternum, which was not deemed a sufficient cause for rejection. The form of declaration referred to was this:

"Navy Yard, Philada., May, 1853.

"I hereby certify that my health is robust; I am free from scrofula, consumption and other hereditary diseases; that I am not suffering from epilepsy, stricture, rupture, or other complaints and injuries, and feel myself able to perform any physical duties required of me as an engineer. All my senses are perfect."

To the physical as well as mental qualifications of engineers, great importance is attached, as their duties are very arduous. They have to undergo great exposure to heat about the engines and fires; they labor hard—watch long and frequently—have use for the nicest and most accurate sense, especially of sight and hearing, in the detection of defective machinery, and are more constantly in active service, it is thought, than any other grade of officers. Acting on a knowledge of these facts, I was under the unpleasant necessity of rejecting three out of thirty-one for defective eyes: one person had a perfect opacity of the left one, and two of them were near-sighted. Nevertheless, one of these procured influence enough to be examined professionally. If, then, he should get into service, and from inability to see a flaw in a steam boiler or other machinery, a disaster should happen, let not the blame rest on his medical inspector.

Of candidates for entrance into the medical corps of the navy, physical perfection is also required; and besides undergoing a satisfactory examination to ascertain that, each candidate has to give a certificate similar to the one quoted. That all persons interested may be fully aware of the requirements demanded, I will state that no one is professionally examined before he has undergone a satisfactory personal inspection, and signed this certificate of "Physical Capacity."

"*Philadelphia*, 1853.

"I declare on honor, that my health at this time is good and robust, and to the best of my knowledge and belief I am free from constitutional defects, and without any predisposition to epilepsy, phthisis pulmonalis, gout or chronic disease of any kind. I have neither circocele, stricture of the urethra, hemorrhoids, nor hernia. Each and all my organs of sense are without imperfection.

"*Candidate for the Office of Assistant Surgeon in the Navy of the United States.*"

Had one of the candidates who appeared before the late Board of Naval Surgeons been aware, previously to his leaving home, that such a certificate as this was required, he might have been saved from the fatigue and expense of a very long journey from the West. That other candidates for the corps may not suffer similarly, their attention is called to these facts and to the following "General Order:"

"*Navy Department, December* 13, 1852.

"It being desirable to obtain the best professional ability which may offer, for the Medical Corps of the Navy, the Department directs:

1st. That hereafter annual Boards shall assemble, at such place as may be designated by the Department, about the close of the lecture seasons of the Colleges, for the selection of candidates for admission into the Medical Corps of the Navy.

2d. Each Board will select from qualified candidates, such a number of the best as may be necessary to meet the demands of the service for the following year.

3d. As vacancies occur in the Medical Corps of the Navy, appointments will be made from the qualified candidates in the order of succession in which they may be named by the Board; but no appointment will be given to any such candidate who is over 25 years of age.

4th. No qualified candidates will be held over for appointment after one year, but all such must be re-examined and take position in the class in which they are last examined-

5th. Every candidate for admission will be examined, strictly and carefully, as to his physical capacity for the service, and the Board will make a separate report in each case, which will be forwarded *direct* to the Department, to be placed on file with the testimonials of the candidate. This examination will precede that as to professional qualifications, and no candidate who is not physically qualified will be examined professionally.

6th. In detailing Boards for the examination of Assistant Surgeons for promotion, as far as practicable, a majority of the members who have served on the Board next preceding, will be selected, in order that the relative position of Assistants of the same date, who have been examined at different times, may be more readily determined.

JOHN P. KENNEDY, Secretary of the Navy."

It may be likewise useful to state that candidates for the corps of midshipmen, previously to their admission to the naval school at Annapolis, must undergo a satisfactory physical inspection by two surgeons appointed for the purpose.

With regard to that of ordinary recruits, as seamen and marines, not so much delicacy is observed as with officers. Every man or boy is entirely stripped of his clothes, a close scrutiny is made of his whole person, and every question is put which may serve to detect natural or acquired defects. To enable the inspector to examine without making omissions, it is best for him to write down all leading questions; if he should not, he may fail occasionally, by omitting some, to detect serious defects—to make the government or employers liable to impositions on account of injuries received anterior to entrance into service—and to admit very useless and annoying persons, such as those affected with stricture in the urethra, piles, fistula, neuralgia and incontenence of urine while asleep, which on board a man of war or other crowded vessel is a great nuisance, from the fœtid condition in which the clothes and bedding of the invalid suffering from it are kept. Equal annoyance may be occasioned by the fœtid exhalations given off by the breath or feet of some individuals—and they ought to be rejected, though perfectly able to perform the duties required of them—as they corrupt the air, and prevent others from the performance of theirs. To put the recruit at ease and compose him,—for a young one is very apt to become agitated and nervous,—affability and kindness of manner should be observed; and he should be questioned on general subjects, by which his sanity of mind may be learned, and that his general condition of health anterior to his application for enlistment may be discovered. When he has become composed he should be made to pace backwards and forwards in a spacious room, that the movements of his joints may be observed ; he should stand first on one foot then on the other, raise them to a horizontal position alternately, and cast each one backwards as high as possible. The hands and arms should perform like evolutions ; the shoulder joints, clavicular junctions, elbows, wrists and fingers be inspected, to find out anchylosis and want of proper flexure, from this or luxation. The loss of a thumb or index finger, deformed feet, toes interfering with walking, varicose veins, signs of scorbutic and scrofulous affections, marks of large ulcers, white swelling, carious bones and deficient muscular development, smallness of size disproportioned to age, symptoms of anæmia, unnatural palor and sallowness, palpitations of the heart continued after the candidate's mind is composed, a very frequent, full and strong pulse in the same condition, I consider causes for rejection. But this, in the above instances and others, must be in a measure governed by length of service, mental or professional qualifications, and the specific duties to be done. A man who could not run aloft, up the shrouds, furl a sail, or clamber up a ledge of rocks, and leap beyond an enemy's trench, might make himself very useful below in working a gun or standing guard. The same remarks will apply to the merchant and militia service.

That no oversights or omissions may happen, I recommend system to be observed in examiniug the different parts and organs of every recruit, beginning with his head and descending to his feet ; and that the inspector ascertain the healthy or unhealthy condition of his various tissues. His scalp should be felt, pressure made upon the cranium to discover fractures, depressed and deficient bones ; the hair ought to be raised to find tinea capitis and other affections. The senses should be tested, particularly those of sight and hearing, the mouth and teeth inspected, the chest sounded, the abdomen, in its whole extent, be felt and pressed upon, the recruit made to speak, to find out whether he stammers, stutters, or has unnatural tone of voice; and when stricture of the urethra is suspected, he ought to be required to urinate or to undergo the introduction of a bougie.

But this and other disagreeable examinations are objected to by recruits, deter them from offering for inspection, and should never be wantonly performed. Even the nudity demanded of them is offensive to some, and I have known several to refuse to strip, and decline enlistment before they would submit to it. In examining the groins and genital organs, special care is to be observed that a rupture be not concealed by being thrust up just before examination, and that a chancre or bubo, either forming, or in the last stage of suppuration, may not be overlooked, or a gonorrhœa may not escape notice. In shipping boys and landsmen, who are mostly superabundant, and can be had at all times plentifully, the latter complaints are good reasons for rejection; but in men who are well drilled, or good sailors, who are always scarce, they are not so, and it is better that these should be taken and cured if it can be effected in a short time. By such indulgence very valuable men may be got or retained in service, and kindness and humanity practised to those who have an additional claim on it from their good conduct and great efficiency. But in these cases the inspector must be in a measure governed very much by the certificate he has to sign—for they differ greatly in purport—and it was in consequence of the one used at the Philadelphia rendezvous, when I went on duty there in October, 1845, being too stringent and preventing me from exercising due discretion or indulgence, that I urged its revocation, and had introduced the present one, which simply states that the recruit—setting forth his name and rank—has been examined and found "fit for the naval service," instead of stating that he is free from bodily defects, as is done in the certificate given each marine enlisted on this station. In many instances I have had the alternative of signing what was not true, as regards defects, or of rejecting desirable men; and to avoid this, I have had several times, with the assent of the commander of the guard, to insert exceptions by writing the defects forming them on the printed certificate. In consequence of such cases, the medical examiner may offend the candidate, dissatisfy the person who wants his services, delay the formation of a regiment, or prevent the early equipment of a ship or squadron. For notwithstanding the change effected by me in the certificate at the rendezvous, I rejected between 10 and 11 per cent. of applicants, or nearly 200 out of 1828, examined by me while last on duty there—that is, including some examined by others while I was absent and on a Board of Examiners. The sum total of rejections in 2010 inspected was 209, or more than 10 per cent. Of these cases, eleven were rejected for varicose veins of the legs, the same number for varicocele, twenty-five for circoceles on the left side without exception to the best of knowledge, thirteen had myopia, nine ophthalmia, five blindness of the left eye, ten imperfect forms, fourteen various cutaneous eruptions, eight diseased heart, nine debility from old age, &c., one was ruptured in the right groin, and nine had been in the left one. For this fact we cannot account, unless by ascribing it to the great expansion of the left abdominal rings—from the larger size of the left spermatic veins than that of the right ones.

Besides the complaints and injuries mentioned as just causes of rejection, we may enumerate contagious, pulmonic and cardiac affections generally, acute and chronic rheumatism and ophthalmia, diseases of the chylopoietic viscera, intestinal, herpetic and other cutaneous affections, secondary syphilis, bronchocele, epilepsy, palsy, spinal deformity or disease, intermittent or other fevers, great loss of teeth and caries of them, and malformations of the body and limbs, debility, great emaciation or exorbitant fatness; in fine, *any complaint* or injury which might excuse a man from military duty, or be urged as a reason for his discharge from it or naval service, as it is very common for such men to get into it, to become dissatisfied and procure their return home or discharge because of these defects.

As has been shown above, of the recruits rejected by me, the largest number were affected with circocele of the left side, varicocele, varicose veins of legs, ophthalmia, myopia, and cutaneous eruptions. Of all examined, whether passed or not, a catalogue was kept and memoranda made, especially of those rejected, as after the lapse of a long time, and when it seemed probable that they were forgotten, some would make another attempt to get into service. Notes upon such persons are not only useful to the one who takes them, but to the officers who succeed or relieve him. These being unsuspecting and uninformed respecting defective applicants, might allow some to be enlisted who might prove incumbrances and nuisances, and utterly worthless, as well from ill health and injury as from bad habits, especially that of confirmed drunkenness, which may have not only undermined their constitutions, but rendered them unfit for any kind of service, by the irregularities it produces, and by the constant tendency it gives to violations of discipline. Concerning the utility of memoranda being kept it may be proper to add, that they afford good statistics of the height, age and diseases of recruits; and moreover enable the inspectors to identify those who have already been enrolled, have failed to enter service at the expiration of their liberty, or, having done so, have deserted or been dismissed as invalids, and endeavor to get re-enlisted for the sake of another bounty or advance of wages. Two instances of such attempts at imposition have come under my own knowledge on this station; and in both of them my recollection of the impostors, assisted by my notes, enabled me positively to recognize them and prevent imposition.

